# Are physicians in primary health care able to recognize pulmonary fibrosis?

**DOI:** 10.1080/20018525.2017.1290339

**Published:** 2017-02-20

**Authors:** Minna Purokivi, Ulla Hodgson, Marjukka Myllärniemi, Eija-Riitta Salomaa, Riitta Kaarteenaho

**Affiliations:** ^a^ Center for Medicine and Clinical Research, Division of Respiratory Medicine, Kuopio University Hospital, Kuopio, Finland; ^b^ Department of Pulmonary Medicine, Heart and Lung Center, Helsinki, Finland; ^c^ Department of Clinical Medicine and Transplantation Laboratory and Helsinki University Hospital, University of Helsinki, Helsinki, Finland; ^d^ University of Turku, Turku, Finland; ^e^ Unit of Medicine and Clinical Research, Pulmonary Division, University of Eastern Finland, Kuopio, Finland; ^f^ Respiratory Medicine, Internal Medicine Research Unit, Medical Research Center Oulu, Oulu University Hospital and University of Oulu, Oulu, Finland

**Keywords:** Referral letter, idiopathic pulmonary fibrosis, interstitial lung disease, primary care, tertiary care, registry

## Abstract

**Background**: The early diagnosis of idiopathic pulmonary fibrosis (IPF) has become increasingly important due to evolving treatment options. IPF patients experience a significant delay in receiving an accurate diagnosis, thus delayed access to tertiary care is associated with higher mortality independently from disease severity.

**Objective**: The aims were to evaluate whether there had been a delay in the referral process, and to determine whether the referring doctors had suspected IPF or other interstitial lung disease (ILD) already during the time of referral.

**Methods**: Ninety-five referral letters of patients with IPF identified from the FinnishIPF registry were evaluated with respect to time of referral, referring unit, grounds for referral, symptoms, smoking status, occupational history, clinical examinations, co-morbidities, medication, radiological findings and lung function.

**Results**: Fifty-nine percent of referral letters originated from primary public health care. The time from symptom onset to referral was reported in 60% of cases, mean time being 1.5 (0.8–2.3) (95%CI) years. The main reason for referral was a suspicion of interstitial lung disease (ILD) (63%); changes in chest X-ray were one reason for referring in 53% of cases. Lung auscultation was reported in 70% and inspiratory crackles in 52% of referral letters.

**Conclusions**: Primary care doctors suspected lung fibrosis early in the course of disease. Lung auscultation and chest X-rays were the most common investigational abnormalities in the referrals. Providing general practitioners with more information of ILDs might shorten the delay from symptom onset to referral.

## Introduction

Idiopathic pulmonary fibrosis (IPF), the most common of the idiopathic interstitial pneumonias, is a progressive lung disease with a survival rate comparable to lung cancer.[1] Though the incidence of IPF has been rising in recent decades, it still seems to be an under-recognized disease in public health care.[2,3] It has been shown recently that the patients with IPF commonly experience significant delays in receiving an accurate diagnosis, the mean delay from symptom onset to diagnosis being 2.2 years.[4,5] This is of special concern, since the delayed access to tertiary health care has been shown to be associated with a higher mortality rate irrespective of the disease severity.[5]

The early referral and diagnostics have become even more important than previously since new treatment options for IPF have become available. Today, there are two disease progression delaying drugs, i.e. pirfenidone and nintedanib, on the market,[6–8] but the only curative treatment for IPF is lung transplantation. The evaluation for lung transplantation should be carried out early in the course of the disease to guarantee a better outcome for those suitable for this procedure.[9,10]

There are several reasons why there may be a delay in referring patients with suspicion of IPF to a tertiary center. For example, the patients may wait for months (or even years) before consulting their primary care doctor due to cough and dyspnea in exertion, the most common symptoms of IPF.[11] Secondly, the suspicion of this rare disease may be missed by physicians working in primary health care, and recognition of its main clinical sign, velcro-like crackles in lung auscultation, may be overlooked.[12] Thirdly, referring to the tertiary care may be complicated by shortcomings in the referral letter, leading to delay in arranging an appointment and even to inappropriate examinations.

In general, the referral letter is an important document; it should contain clinical background information, data from performed diagnostic tests and specify the purpose of the referral to the specialist in tertiary care.[13–17] For this study, a group of senior consultants from four university hospitals evaluated the contents of patients’ referral letters included in the FinnishIPF registry. These patients had been referred to the tertiary care respiratory clinics due to a suspicion of respiratory problems. The aims of this study were to evaluate whether there had been a delay in the referral process, and to determine whether the referring doctors had suspected that their patient had IPF or other interstitial lung disease (ILD) already during the time of referral. The presence of specific findings known to contribute to respiratory health, e.g. smoking, occupational history, co-morbidities, previous medication, and information of performed diagnostic tests, was also evaluated. This retrospective analysis may be helpful in planning future educational interventions for the physicians working in the primary care.

## Materials and methods

### Study design

Referral letters of 95 subjects collected from the FinnishIPF registry, whose IPF diagnosis had been re-evaluated according to recent classification, were scrutinized by four senior consultants of respiratory medicine.[18,19] The subjects had been originally referred to tertiary care due to respiratory problems, and they had been examined in one of the following University hospitals in Finland: Helsinki, Kuopio, Oulu or Turku. A structural data acquisition form was utilized in data collection.

### Data acquisition form

A two-step Delphi exercise was performed prior to devising the data acquisition form which would be used in the evaluation of the quality of the referral letters. Ten senior consultants of respiratory medicine in a tertiary center were asked which details they found to be the most important in a referral letter for a respiratory clinic. The data acquisition form was then modified by the present authors to focus especially on the suspicion of ILD. The usability of the form was tested by the authors with eight referral letters. The collected data concerned time of referral, referring unit/specialty, reason for referring, description of patients’ complaint, smoking status, occupational status, history of exposure, previous examinations (chest X-ray, high resolution computed tomography (HRCT), spirometry, diffusion capacity, laboratory findings, oxygen saturation), co-morbidities, medication, and clinical findings (auscultation of the lungs).

### Ethical considerations

Helsinki University Central Hospital Ethical Committee provided approval for this study, and it has been accepted by the ethical committees of the above-mentioned university hospitals. The National Institute of Health and Welfare has given authorization for patient screening from hospital databases. The study has been performed in accordance with the ethical standards laid down on the 2000 Declaration of Helsinki. All patients have given their written informed consent.

### Statistical analysis

IBM SPSS Statistics version 22 (NY, USA) was used in statistical analyses. Percentages, mean (min-max) and mean (95% confidence interval) were used to describe the data. Paired samples t-test in comparing continuous variables between time-points and chi-square test in comparison of categorical variables were utilized. Correlations were analyzed with Spearman’s correlation coefficient.

## Results

### Origin of referral letters

Referral letters of 91 from 95 subjects were found from hospital registries for data collection. The basic characteristics of the subjects are described in Table 1. Most of the referral letters had been written in public health care by general practitioners working in health care centers (59%) and specialists in tertiary health care (18%). Of the referral letters 22% had arrived from private doctors, mainly from specialist of respiratory medicine (15%), with some from occupational health care (2%).

**Table 1.** Characteristics of the patients with idiopathic pulmonary fibrosis (IPF) and distribution of referral letters between university hospitals.

Number of subjects	91
F/M	39/52
Age^#^	55 (34–89)
FVC^¶^	2.6 (2.1–3.0)
FVC (%predicted)^¶^	73 (50–82)
FEV_1_^¶^	2.1 (1.7–2.5)
FEV_1_ (%predicted)^¶^	75 (55–95)
FEV_1_ /FVC (%predicted)^¶^	0.82 (73–94)
**Referral letters per university hospital**	
Helsinki	35 (39%)
Turku	14 (15%)
Kuopio	18 (20%)
Oulu	24 (26%)

^#^Age (years), FEV_1_% from predicted, FVC% from predicted and FEV% are expressed as mean (min–max). FEV_1_, FVC in l min^–1^ are expressed as mean (95% confidence interval). ^¶^Reference values of Viljanen [20].

### Characteristics of the referral letters

The characteristics of the referral letters are shown in Table 2. Certain variables that may be closely related to evaluation of the origin of ILD such as smoking, occupational factors, co-morbidities and medication, were missing in 17–52% of referral letters. The number of ever-smokers (51%) did not differ between male and female subjects. The patient’s overall ability to function was described in 32% of referral letters.

**Table 2.** Presence of key information in referral letters.

**Overall description of symptoms**	82 (90 %)
Cough	34 (43 %)
Dyspnea	36 (46 %)
Sputum production	5 (6 %)
Hemoptysis	1 (1 %)
Duration of symptoms described	66 (60%)
**Smoking**	
Mentioned in referral letter	53 (58%)
Ever-smokers	27 (51%)
**Occupational history**	
At work during the time of referral	19 (22%)
Retired	62 (70%)
Not known	8 (8%)
**Detailed information of profession**	
Farmer	2 (2%)
Blue collar work	27 (30%)
White collar work	15 (16%)
Information missing	47 (52%)
**Co-morbidities**	
Presence or absence mentioned	74 (81%)
Information missing	17 (19%)
**Previous medication**	
Presence or absence mentioned	63 (69%)
Information missing	28 (31%)
**Clinical findings**	
Inspiratory crackles	46 (50%)
No crackles	16 (18%)
Information missing	29 (32%)
**Objective tests as an attachment**	
Chest X-ray	84 (92%)
High resolution computer tomography (HRCT)	16 (18%)
Spirometry	26 (29%)
Diffusion capacity	4 (4%)
Oxygen saturation	3 (3%)
Laboratory tests	38 (42%)

### Symptoms

The time from the onset of symptoms to referral had been approximated in 60% of referral letters, mean time being 1.5 (0.8–2.3) years. There was no gender-related difference in the delay from symptom onset to referral. An overall description of the symptoms was found in 90% of referral letters, with cough (43%) and dyspnea (46%) being the most common symptoms.

### Clinical findings

The clinical status had been described in 71% of referral letters, usually very briefly. The lung auscultation was described in 70% of referral letters and inspiratory crackles had been detected in every second case.

### Co-morbidities

The presence or absence of co-morbidities was reported in 83% of referral letters. Thirty patients were reported to have one or more chronic diseases. The most common co-morbidities were arterial hypertension (35%), diabetes (20%) and cardiovascular disease (27%). Three patients had gastro-esophageal reflux disease. A previous diagnosis of COPD was reported twice and chronic cardiac failure once.

### Treatment interventions preceding referral

In 18 referral letters one or several treatment trials for respiratory symptoms were described. The treatments used were antibiotics (10 cases), oral corticosteroids (one case), inhaled corticosteroids and/or long-acting beta-agonists or long-acting muscarin receptor antagonist (four cases), short-acting beta-agonists (one case), diuretics (one case), nitrates (one case), and proton pump inhibitor (one case).

### Chest X-ray and spirometry

Some comment about the chest X-ray was attached to 92% of referral letters. The referring doctor had stated that the changes in the chest X-ray was one reason for referring in 53% of referral letters. Twenty-six referral letters (29%) included data of spirometry from referring unit, and in seven cases, the decreased lung function was considered as a reason for referral. The presence of radiological changes in chest X-rays was not associated with the reduction of lung volume.

### Disease severity at the time of referral

To evaluate the disease severity in the time of referral, the first spirometry measured in the respiratory unit of the tertiary clinic was sought for analyses (Table 1). In all, 42% of the patients had normal lung function. Forced vital capacity (FVC) was reduced mildly (65–79% from predicted) in 34% and moderately (45–64% from predicted) in 16% of cases.[21] The mild reduction of lung function was more common in male than in female subjects, (20 versus 9, *p* = 0.024). Twenty-six referral letters (29%) included data of spirometry from referring unit, and therefore we conducted a comparison of these values with those assessed at the first evaluation at the tertiary center; we could detect no evidence of significant disease progression in this subgroup, i.e. their condition had not deteriorated due to the delay while waiting for their visit to tertiary care consultation.

### Reason for referral to tertiary center

The suspicion of ILD in particular was mentioned in 63% of referral letters. A suspicion that there might be some illness other than ILD was described in 16% of referral letters. In eight cases, an occupational lung disease was suspected. The researchers estimated in their evaluation that the main reasons for referral were specific symptoms in 50% of cases including cough (25%) and/or dyspnea (34%), and radiological findings in 23% of the cases. Inspiratory crackles as a reason for referral were mentioned in 16% of referral letters. The mean time from referral to the first visit in the tertiary center was 56 days (range 0 to 216 days).

### Quality score for the required minimum data

A quality score for the minimum data requirement for a good referral letter in IPF suspicion was created. The score consisted of five variables which could be evaluated in primary care: description of smoking history, patient’s symptoms, findings from lung auscultation, chest X-ray picture and spirometry. Each variable produced one point for the score. In 31% of letters two of the required variables were mentioned, in 31% three variables, in 14% four variables but in only 6% were all five variables found. The quality of referral letters did not depend on their origin. The quality score did not correlate with the disease severity defined according to FVC, smoking history, length of the symptoms, and delay from referral date to the first visit in primary care or with the actual suspicion of any parenchymal lung disease mentioned in the referral letter.

## Discussion

As far as we are aware, this is the first study that has evaluated the referral practice of IPF patients to tertiary care through referral letters. This is of special interest, since the early diagnosis of IPF has become more important due to development of new, disease-specific treatment options for this deadly condition. In the present patient population the mean delay from symptom onset to time of referral was still 1.5 years, paralleling the findings of previous studies.[4,5,21] Reasons for this delay should be recognized and rectified if the aim is to reduce IPF related mortality.

In this study, more than half of the referral letters originated from primary health care, suggesting that the physicians in primary health care seem to suspect ILDs quite well. However, certain shortcomings in the referral letters were regrettably common. The content of the anamnesis was often inadequate. In concordance with previous studies, a history of smoking was missing in 58% of cases though it is one of the most important etiological causes of chronic lung diseases.[11] Similarly, information of the occupational history and an evaluation of exposures during working life were missing in more than half of the referral letters. The referring doctor may ignore the occupational history due to the advanced age of patient but it is crucial to remember that some exposures may represent a health hazard only after a long delay, e.g. asbestos.[22] Information about previous medication is of importance when studying lung infiltrates as many pharmacological compounds are known to evoke changes in lung parenchyma. Unfortunately, this information was mostly absent, as reported also previously.[14] The overall ability to function, which is an important factor when planning the clinical examinations for a patient in tertiary care, was missing in two out of every three referral letters. The referral letter should provide a basis for selection of further diagnostic tests, and these kinds of shortcomings may lead to inappropriate examinations that cause both inconvenience to the patient and unnecessary expense.

The most typical symptoms of IPF in the early stage of the disease are dyspnea and cough, as was the case also in the present sample of patients.[11,12,23] As reviewed recently by Lee et al. [24], dyspnea predicts both survival and mortality of IPF patients, whereas cough has been reported as an independent predictor of disease progression, and time-to-lung transplantation or death.[24,25] Both of these symptoms are common in adults, especially in the aging population, and therefore, their importance may be underestimated, leading to an unnecessary diagnostic delay. In this study population several patients had undergone at least one treatment trial before they were referred to tertiary care for further investigations. In the differential diagnosis of prolonged cough or dyspnea of middle aged or elderly individuals, IPF as well as other ILDs should be borne in mind.[26]

Clinical findings were missing from a third of the referral letters, though auscultation of lungs is an inexpensive and useful diagnostic procedure available in all primary health care units. According to the literature, there seems to be a trend that physicians underestimate the value of classical clinical examinations in diagnostics.[27] It is noteworthy that inspiratory crackles can be heard from the lower lung zones already in the early course of IPF.[28] Though the crackles are more common in elderly patients,[29] the presence of bilateral fine crackles should trigger a more detailed anamnesis, with possible further investigations, such as chest X-ray and spirometry, undertaken in the primary care.

There was considerable variation in the quality of the reporting of investigations conducted preceding the referral to tertiary care.[27] There were comments about findings of the chest X-ray in almost every referral letter of this study, and the referring doctor had named the radiological changes as a reason for referral in every second case. It has been reported that radiological changes can be present in early stages of the disease, even if the spirometry still is in normal limits.[30] The numbers of spirometries performed in primary care were found to be surprisingly low, although almost every health center in Finland has the equipment and capability to perform those tests. Previously, the quality of primary care spirometry in Finland has been assessed as good.[31] However, primary care doctors may need more training on the use of spirometry in differential diagnosis of respiratory diseases. Though the restrictive ventilation pattern may be missing in the early stage of the disease, the follow-up of spirometry may be valuable in assessing the disease progression in an individual patient.

In the majority of the referral letters the presence of co-morbidities was noted. Paralleling a recent Danish study,[32] arterial hypertension, cardiovascular diseases and diabetes were the most common co-morbidities. The presence of these chronic conditions may act as a confusing factor which could well lead to delayed suspicion that there is a different lung disease.

In line with previous findings of larger IPF cohorts, most of our subjects had normal or only mildly reduced lung function at the time of admission to tertiary care.[32,33] A slightly reduced lung function was more common among men. The current data do not reveal the reason for this difference, which may be attributable to the failure of the physicians to describe their patients smoking habits in the referral letter. For only a small subset of patients a spirometry evaluation had also been conducted in primary care. In this subset IPF had not deteriorated significantly during the delay from the time of referring to the first visit to tertiary care.

In this cohort, the mean delay from the referral date to the first visit in tertiary care was 56 days. Suspicion of parenchymal lung disease, such as IPF, is not considered as a reason for an urgent referral either in Finland or internationally. According to the current national directions this patient group should be evaluated within three months of referral, which was achieved in 91% of the current cases.

According to the present results primary care general practitioners suspected ILDs rather well. However, in 49% of referral letters, three out of five or even more of the key items of information (description of smoking history, patient’s symptoms, lung auscultation, chest X-ray finding and spirometry) were missing. This shows that the referring doctors’ readiness to perform diagnostic tests, especially spirometry, has not improved in the last 10 years.[14] In addition, improvements are still needed in the reporting of anamnesis and basic clinical examinations. Education of primary care doctors has been shown to improve quality of referral letters and disease recognition.[34] A better interaction between specialists and referring physicians, such as regular meetings, would be one way to reduce the number of inappropriate and incomplete referrals.[16] However, simply providing guidelines does not seem to improve the referral quality.[17] In the case of rather rare diseases, such as IPF, the updating of guidelines may not reach the primary care general practitioner, who struggles with a wide range of problems in his/her routine practice.

In conclusion, primary care physicians suspect lung fibrosis early in the course of disease, and were able to utilize the appropriate diagnostic procedures. Nonetheless, shortcomings in referral letters were common. Inspiratory crackles in lung auscultation together with prolonged dyspnea and/or cough should alert the general practitioner to refer the patient for a consultation in tertiary care. Provision of more easy-to-understand information about ILDs to the general public might shorten the delay from symptom onset to referral, since it seems to be largely attributable to the inability of the patients to recognize the seriousness of these common symptoms.
